# Genome-wide identification of the *GATA* gene family in melon (*Cucumis melo*) and analysis of their expression characteristics under biotic and abiotic stresses

**DOI:** 10.3389/fpls.2024.1462924

**Published:** 2024-09-13

**Authors:** Ling Zheng, Lin Tang, Jinbo Li

**Affiliations:** Department of Biology, Luoyang Normal University, Luoyang, Henan, China

**Keywords:** *Cucumis melo*, GATA gene family, drought stress, heavy metal lead stress, *Fusarium* wilt infection

## Abstract

GATA transcription factors are an important class of transcription factors in plants, known for their roles in tissue development, signal transduction, and responses to biotic and abiotic stresses. To date, there have been no reports on the *GATA* gene family in melon (*Cucumis melo*). In this study, 24 *CmGATA* genes were identified from the melon genome. These family members exhibit significant differences in protein length, molecular weight, and theoretical isoelectric point and are primarily located in the nucleus. Based on the classification of *Arabidopsis thaliana GATA* members, the phylogenetic tree divided them into four groups: group I, group II, group III, and group IV, containing 10, 8, 4, and 2 genes, respectively. Notably, *CmGATA* genes within the same group have highly conserved protein motifs and similar exon–intron structures. The *CmGATA* family members are unevenly distributed across 10 chromosomes, with six pairs of segmentally duplicated genes and one pair of tandemly duplicated genes, suggesting that gene duplication may be the primary factor in the expansion of the *CmGATA* family. Melon shares 21, 4, 38, and 34 pairs of homologous genes with *A. thaliana*, *Oryza sativa*, *Cucumis sativus*, and *Citrullus lanatus*, respectively. The promoter regions are enriched with various *cis*-acting elements related to growth and development (eight types), hormone regulation (nine types), and stress responses (six types). Expression patterns indicate that different CmGATA family members are significantly expressed in seeds, roots, stems, leaves, tendrils, mesocarp, and epicarp, exhibiting distinct tissue-specific expression characteristics. Quantitative fluorescence analysis revealed that five genes, *CmGATA3*, *CmGATA7*, *CmGATA16*, *CmGATA22*, and *CmGATA24*, may be highly active under 48-h drought stress, while *CmGATA1* and *CmGATA22* may enhance melon resistance to heavy metal lead stress. Additionally, *CmGATA22* and *CmGATA24* are suggested to regulate melon resistance to *Fusarium* wilt infection. *CmGATA22* appears to comprehensively regulate melon responses to both biotic and abiotic stresses. Lastly, potential protein interaction networks were predicted for the CmGATA family members, identifying *CmGATA8* as a potential hub gene and predicting 2,230 target genes with enriched GO functions. This study preliminarily explores the expression characteristics of *CmGATA* genes under drought stress, heavy metal lead stress, and *Fusarium* wilt infection, providing a theoretical foundation for molecular mechanisms in melon improvement and stress resistance.

## Introduction

Plants frequently encounter biotic and abiotic stresses, including drought, extreme temperatures, salinity, and pest infestations, during their growth and development (Mittler et al., 2022; Hilty et al., 2021). Over evolutionary timescales, plants have developed various defensive strategies to mitigate these stressors’ impacts, including adjusting stomatal sizes to cope with high temperatures (Niu and Xiang, 2018), balancing ion levels to counteract salt stress (Liang et al., 2018), and developing protective structures such as cuticles, waxy coatings, and cork layers to prevent invasions by pests and pathogens (Mitchell et al., 2016; Koch et al., 2016). Transcription factors (TFs), proteins that bind to specific DNA sequences and regulate gene expression, play pivotal roles in these processes (Strader et al., 2022; Javed et al., 2020). They influence plant signal transduction, growth, development, and responses to biotic and abiotic stresses (Strader et al., 2022; Javed et al., 2020). Serving as central components of gene expression regulatory networks, they control various physiological and developmental processes in cells by either activating or inhibiting transcription (Ng et al., 2018; Romani and Moreno, 2021).

Extensive research has been conducted on various transcription factor families in plants, such as WRKY (Jiang et al., 2017), AP2/ERF (Feng et al., 2020), MYB (Wang et al., 2021), bHLH (Guo et al., 2021a), and bZIP (Han et al., 2023), all of which have been functionally characterized. In agricultural production, understanding the regulatory mechanisms of these transcription factors can significantly improve crop quality, resilience, and yield.

GATA transcription factors are named for their binding sites containing the GATA sequence and are prevalent in eukaryotes, playing crucial roles in cellular differentiation, development, and physiological function (Block and Shapira, 2015; Schröder et al., 2023). The DNA-binding domain of these factors consists of two zinc fingers (C4-type zinc fingers) located in the middle and at the C-terminal (An et al., 2020). These structures recognize and bind to specific DNA sequences (usually GATA sequences), thereby regulating the expression of target genes (Romano and Miccio, 2020). In addition to the zinc finger domains, GATA transcription factors often include N-terminal and C-terminal regions that may contain domains with transcriptional activation or inhibition functions (An et al., 2020). The highly conserved nature of the zinc finger domains throughout evolution underscores their essential roles in fundamental biological functions (Zhang et al., 2018, 2019).

In plants, GATA transcription factors constitute an important family that regulates growth, development, and responses to various environmental stresses. Currently, the GATA family has been studied and reported in various plants, including *A. thaliana* (30) (Reyes et al., 2004), *O. sativa* (28) (Reyes et al., 2004), *Glycine max* (64) (Zhang et al., 2015), *Solanum tuberosum* (49) (Yu et al., 2022), *Triticum aestivum* (79) (Feng et al., 2022), and *Pyrus bretschneideri* (32) (Manzoor et al., 2021). Among these, *A. thaliana* has been studied the earliest and has the most comprehensively identified gene functions within the GATA family. The phylogenetic analysis of GATA family members in *A. thaliana* classifies them into four groups: I, II, III, and IV (An et al., 2020). These factors are instrumental in light signal transduction, regulating the expression of photoreceptor genes and modulating plant responses to light (Zhang et al., 2020). They play significant roles in photomorphogenesis and chlorophyll biosynthesis (Du et al., 2022). For example, *GATA21* and *GATA22* can promote seed growth and development under light conditions (Gangappa and Chattopadhyay, 2013). Fungi regulate iron absorption through *GATA* genes (Haas et al., 1999). In beans, transgenic lines of *PvGATA28* show remarkable tolerance to abiotic stresses (Abdulla et al., 2024). In maize, *ZmGATA37* enhances resistance to biotic stresses (Hu et al., 2023). In potatoes, *StGATA9* increases resistance to bacterial wilt (Yu et al., 2022). A deeper understanding of the functions and mechanisms of GATA transcription factors can shed light on plant stress adaptation strategies, providing new approaches and methods for breeding stress-resistant crops.

Melon (*Cucumis melo* L.), a member of the Cucurbitaceae family, is economically important for its juicy fruit, which is rich in carbohydrates and nutrients. During cultivation, melons often face various environmental stresses, which can reduce fruit yield and quality. In Luoyang, Henan Province, melon plants frequently suffer from *Fusarium* wilt infections, leading to widespread plant death, and drought conditions during the fruit development phase in summer. Additionally, local industrial development has led to heavy metal contamination in some soils. However, there has been no research reported on the GATA transcription factor family in melon. Therefore, this study aims to identify members of the melon GATA transcription factor family and perform bioinformatic analyses, including analyses of protein physicochemical properties, phylogenetics, conserved motifs, gene structure, gene duplication, cis-acting elements, and expression patterns. Furthermore, the ‘Super Sweet White Sugar Pot’ melon variety was subjected to drought stress, heavy metal lead stress, and *Fusarium* wilt infection treatments, with quantitative fluorescence expression analysis conducted on ten selected *CmGATA* genes. This research provides insights into the structure and function of *CmGATA* genes.

## Materials and methods

### Identification of GATA family members in melon

The melon (*C. melo*) DHL92 v4.0 genome was downloaded from the CuGenDB database (http://cucurbitgenomics.org/) for the identification of *GATA* genes (Garcia-Mas et al., 2012). Using the Hidden Markov Model (PF00320), the melon protein database was searched with the HMMER tool, retaining genes with E-values ≤ 1e^− 5^ (Potter et al., 2018). Additionally, the BLAST tool was used to compare these sequences against *A. thaliana* GATA protein sequences, removing duplicates and retaining genes with E-values ≤1e^−5^ (Ye et al., 2006). Lastly, using the same methodology, 26 and 25 *GATA* family members were identified in cucumber and watermelon, respectively. The genomic data for cucumber (*C. sativus*) and watermelon (*C. lanatus*) were obtained from the Ensembl Plants database (http://plants.ensembl.org/index.html). Ultimately, 24 GATA genes were identified in the melon genome. Protein physicochemical properties were analyzed using ExPASy (http://web.expasy.org/protparam/) (Wilkins et al., 1999), and subcellular localization was analyzed using WoLF PSORT II (https://www.genscript.com/wolf-psort.html?src=leftbar) (Horton et al., 2007).

### Phylogenetic tree construction and classification

There were 30 protein sequences of the *A. thaliana GATA* family that were downloaded from the UniProt database (https://www.uniprot.org/) (UniProt Consortium, 2021). To compare the evolutionary relationships and identify the subfamilies, the putative *GATA*s from *C. melo*, *A. thaliana*, *C. sativus*, and *C. lanatus* were used to construct the phylogenetic tree using the Neighbor-Joining (NJ) method, with 1,000 bootstrap replications, employing the Poisson correction model and pairwise deletion (Kumar et al., 2016). Based on the phylogenetic tree, the members were classified into four groups, group I, group II, group III, and group IV, and the tree was visualized using iTOL (https://itol.embl.de/) (Letunic and Bork, 2021).

### Conserved motifs, domains, and gene structures

The 24 CmGATA protein sequences were analyzed for motifs using the MEME tool (http://meme-suite.org/tools/meme) with a default setting of 10 motifs (Bailey et al., 2009). The positions and numbers of GATA domains within the *CmGATA* family protein sequences were identified using the NCBI CDD tool (https://www.ncbi.nlm.nih.gov/cdd/) (Marchler-Bauer et al., 2015). Exon and intron positions and numbers in the *CmGATA* genes were computed from the melon genome data. Finally, phylogenetic trees, motifs, domains, and gene structures of the CmGATA family members were visualized using TBtools software (Chen et al., 2020).

### Gene localization, duplication, and Ka/Ks values

The gene distribution of *CmGATA* members on chromosomes was plotted using TBtools software. Segmental and tandem duplication analyses of *CmGATA* members were performed using MCScanX, and chromosome collinearity was visualized using Circos (Wang et al., 2012; Krzywinski et al., 2009). Orthologous gene pairs among *C. melo*, *A. thaliana*, *O. sativa*, *C. sativus*, and *C. lanatus GATA* genes were identified. The non-synonymous (*Ka*) and synonymous (*Ks*) substitution rates and Ka/Ks values were calculated using the KaKs Calculator tool (Zhang et al., 2006).

### Promoter annotation

The 2,000-bp (base pair) upstream promoter sequences of the 24 *CmGATA* genes were extracted for *cis*-acting element annotation using the PlantCARE online tool (http://bioinformatics.psb.ugent.be/webtools/plantcare/html/) (Lescot et al., 2002). The distribution of cis-acting elements related to abiotic and biotic stresses, phytohormone responsiveness, and plant growth and development was mapped (Zhang et al., 2024).

### Expression patterns

The expression patterns of the *CmGATA* family members in seven tissues (seeds, roots, stems, leaves, tendrils, mesocarp, and epicarp) were analyzed using FPKM (Fragments Per Kilobase of exon model per Million mapped fragments) values obtained from the melon transcriptome database (http://cucurbitgenomics.org/rnaseq/home) (Yu et al., 2023). A coefficient of greater than or equal to 0.8 was considered significant expression.

### Quantitative fluorescence

Seeds of the ‘Super Sweet White Sugar Pot’ melon variety were soaked, germinated, and sown. Plants at the three-leaf stage were cultured in a greenhouse under a 16/8-h light/dark cycle at 28°C/18°C. *Fusarium oxysporum* was isolated from infected melon plants. To prepare a spore suspension at a concentration of 1 × 10^^8^ spores/mL, the root drenching method was employed for inoculation, with each plant receiving 10 mL of the suspension. Samples were collected from the roots, mid-stem, and the first true leaf at 0 h, 12 h, 24 h, and 48 h post-inoculation. Plants were also subjected to drought stress using a 15% PEG 6000 solution and to heavy metal lead stress by applying a Pb(NO_3_)_2_ solution adjusted to a concentration of 2,000 mg/kg Pb ions in the potting soil. Leaf samples were collected at the same time points. All treatments were biologically replicated three times, and samples were immediately stored at −80°C.

Quantitative real-time PCR (qRT-PCR) was used to explore the expression levels of 10 selected *CmGATA* genes posttreatment (leaves; three biological replicates) and postinfection (roots, stems, and leaves; three biological replicates) (Singh et al., 1998). Total RNA from all samples was extracted using the Spectrum™ Plant Total RNA Kit (Merck KGaA) and assessed with a NanoDrop 2000. First-strand cDNA was synthesized using the SweScript All-in-One First-strand cDNA Synthesis Kit (TRANS, G3337). qPCR was performed on a CFX96 real-time PCR Detection System (Bio-Rad, Hercules, CA) using 2× SYBR Green qPCR Master Mix (no ROX) (TRANS, G3320). The cycling conditions were as follows: 95°C for 30 s, followed by 40 cycles of 95°C for 15 s and 60°C for 30 seconds. XM_008449635.3 was used as the reference gene, and relative gene expression levels were calculated using the 2^−ΔΔCT^ method (**Supplementary Table 1**) (Livak and Schmittgen, 2001).

### Prediction of target genes and protein interactions

The protein sequences of CmGATA family members were uploaded to the STRING database (https://string-db.org/) to perform node comparisons, predicting the interactions among CmGATA members based on *A. thaliana* protein interaction data (Szklarczyk et al., 2023). The binding motif map of *A. thaliana* GATA12 transcription factor (MA1015.1) was obtained from the JASPAR Plantae database (https://jaspar.elixir.no/search?q=&collection=CORE&tax_group=plants) (Castro-Mondragon et al., 2022). Subsequently, the 2,000-bp promoter sequences of all genes in the melon genome were extracted, and genes that bind to the GATA12 transcription factor were detected using the Motif FIMO tool (https://meme-suite.org/meme/). Finally, the structural domains of target genes were predicted based on the PFAM database (Mistry et al., 2021), and KEGG (Kyoto Encyclopedia of Genes and Genomes) and GO (Gene Ontology) enrichment analyses of target genes were conducted using OmicShare Tools (https://www.omicshare.com/tools).

### SSR loci and microRNA prediction

SSR (simple sequence repeat) loci in the 24 GATA gene sequences of melon were identified using the Batch SSR Finder tool (Song et al., 2021). The microRNA (miRNA) sequences of melon were obtained from the PNRD (Plant Non-Coding RNA Database: https://structuralbiology.cau.edu.cn/). The psRNATarget (https://www.zhaolab.org/psRNATarget) online tool was used to predict miRNAs of the CmGATA genes (Fu et al., 2024).

### Prediction of protein tertiary structure

The SWISS-MODEL database was used to predict the tertiary structures of the 24 GATA protein sequences in melon (Waterhouse et al., 2018).

## Results analysis

### Analysis of *CmGATA* member characteristics

There were 24 *GATA* genes identified in the melon genome, which were renamed *CmGATA1* to *CmGATA24* based on their chromosomal distribution and sequence order. Significant variations in the physicochemical properties of CmGATA members were observed (**Supplementary Table 2**). The number of amino acids in CmGATA members ranged from 139 aa (amino acid) (CmGATA24) to 565 aa (CmGATA16), and the molecular weight ranged from 15,071.06 Da (Dalton) (CmGATA24) to 63,040.70 Da (CmGATA16). The theoretical isoelectric points ranged from 5.03 to 9.71, with 14 members having a pI less than 7 and 10 members having a pI greater than 7. All CmGATA members were identified as hydrophilic proteins. Subcellular localization results revealed that *CmGATA5* and *CmGATA19* lacked start codons; thus, subcellular localization could not be predicted. The majority of other members were primarily located in the nucleus.

### Phylogenetic analysis of *CmGATA*

**Figure 1:**
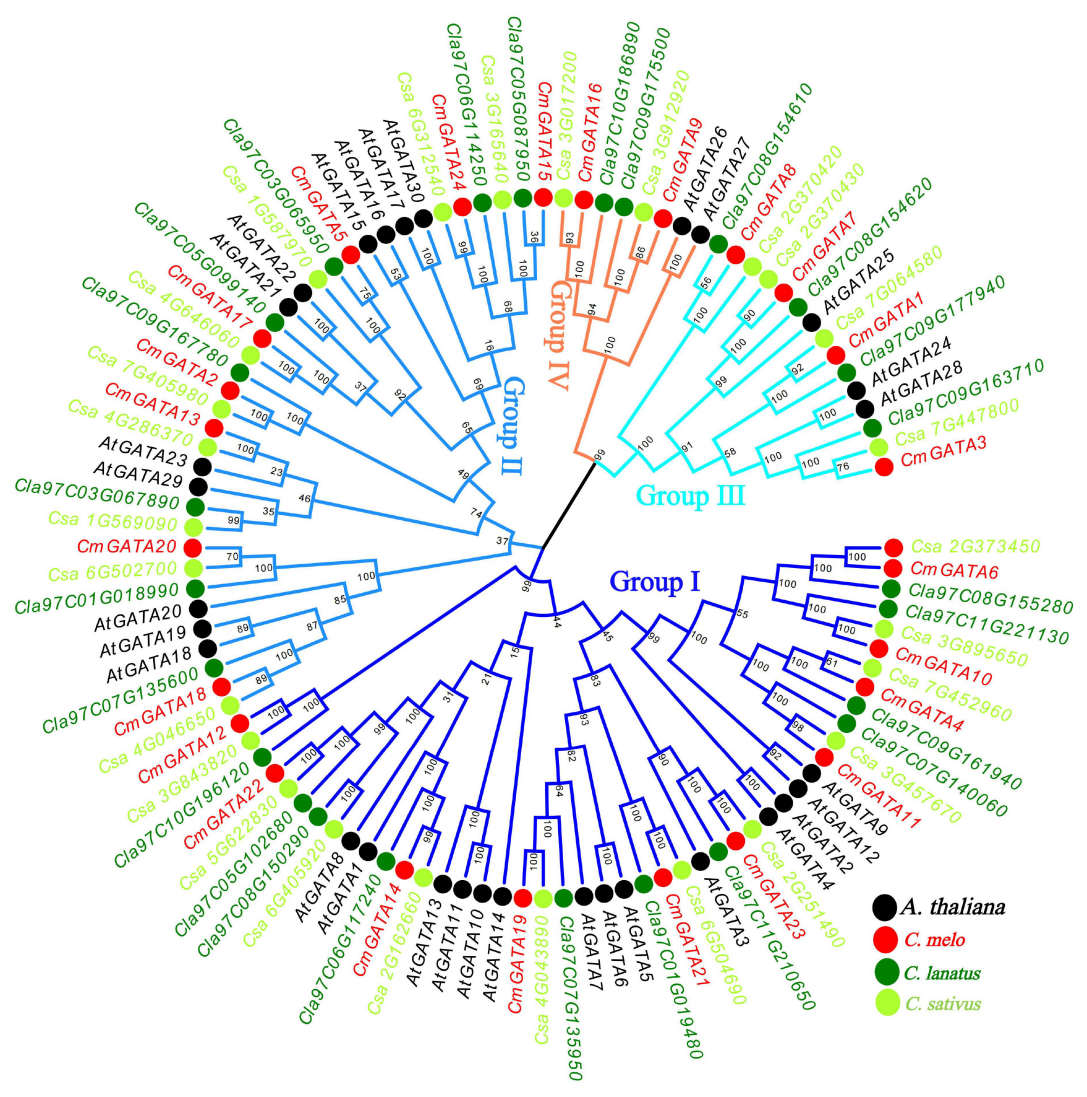
Neighbor-joining phylogenetic tree of GATA family members in *A. thaliana*, *C. melo*, *C. sativus*, and *C. lanatus*. The four branch colors of the evolutionary tree represent the four groups. The solid circles in four different colors represent the genes of the four species, respectively.

The MEGA 7 tool was used to construct a Neighbor-Joining phylogenetic tree of GATA family members from four species: *C. melo*, *A. thaliana*, *C. sativus*, and *C. lanatus* (**Figure 1**). Based on the classification of *AtGATA* members, the *GATA* genes were divided into four groups: group I, group II, group III, and group IV. Groups I to IV contained 10, 8, 4, and 2 *CmGATA* genes, respectively. Notably, the *GATA* genes corresponding to melon, cucumber, and watermelon in these four groups were highly clustered together, suggesting a lower degree of evolutionary divergence among these three species. Furthermore, domain analysis revealed varying degrees of conservation among the CmGATA proteins within these groups (**Supplementary Figure 1**). Groups I, II, and III exhibited lower overall sequence conservation, whereas group IV showed higher conservation. It is also noteworthy that all CmGATA members demonstrated high conservation at the amino acid residues in the LCNACG site.

### Analysis of conserved motifs, domains, and gene structures in *CmGATA* members

**Figure 2:**
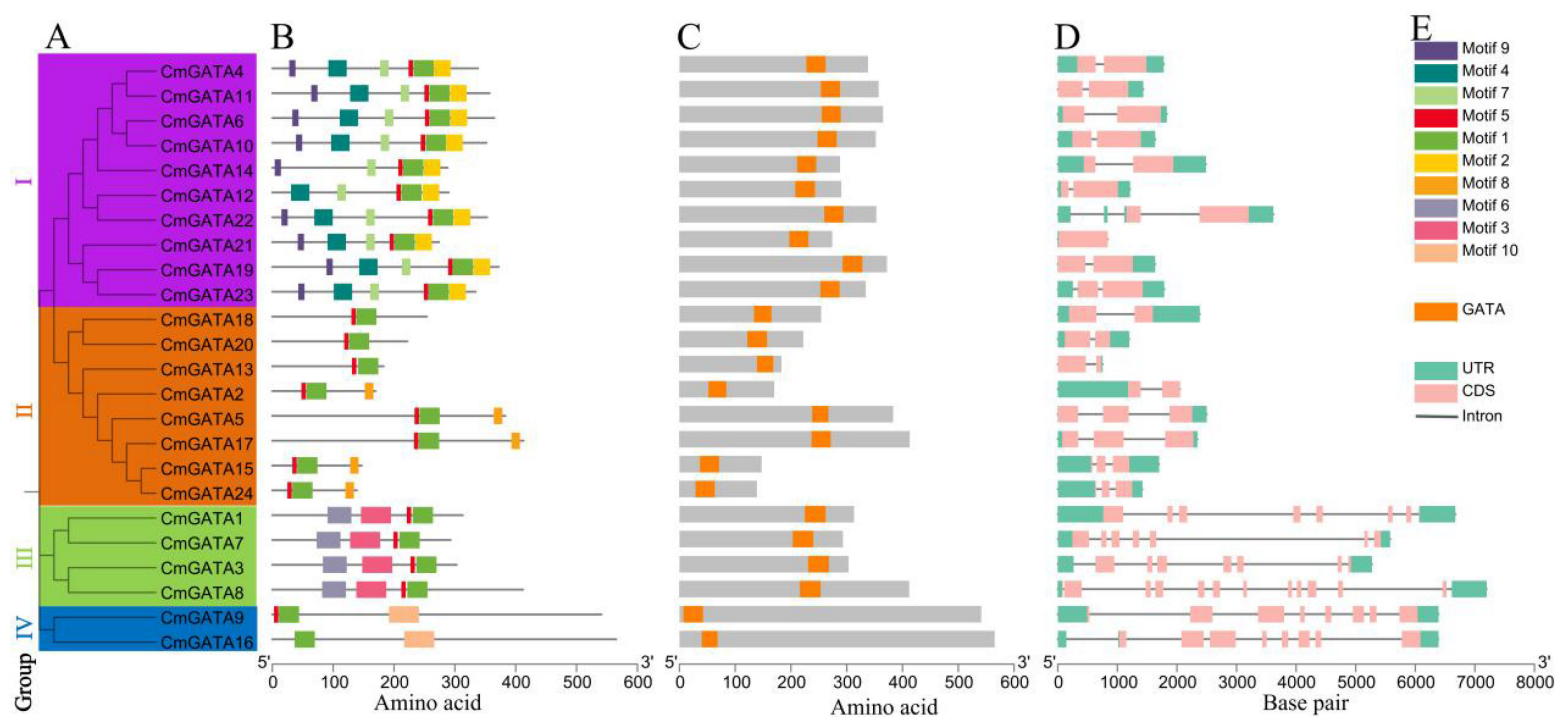
Analysis of the phylogenetic tree, conserved motifs, domains, and gene structures of *CmGATA* family members. **(A)** Neighbor-Joining phylogenetic tree. **(B)** Conserved protein motifs. **(C)** Distribution of GATA domain positions. **(D)** Distribution of exons and introns. **(E)** Rectangle colors and corresponding types.

The phylogenetic tree, conserved motifs, domains, and gene structures of the *CmGATA* family members were integrated and visualized (**Figure 2A**). The clustering results of the *CmGATA* family members on the Neighbor-Joining phylogenetic tree were consistent with those shown in **Figure 1**. Based on the grouping in **Figure 1**, they were divided into four groups (**Figure 2A**). There were 10 motifs annotated in the CmGATA family members, and their types were identified using the NCBI CDD tool (**Supplementary Figure 2**). Of these, motif 1 corresponds to the GATA zinc finger, motif 3 to the CCT motif, and motif 7 to the tify domain, while the other seven motifs do not correspond to specific structural domains. Notably, there is significant variability in the types and numbers of motifs among the members of the four groups (**Figure 2B**). For instance, members of group I primarily consist of motifs 9, 4, 7, 5, 1, and 2. Members of group II mainly contain motifs 5 and 1. Group III members primarily have motifs 6, 3, 5, and 1. Group IV members predominantly consist of motifs 1 and 10. The protein domain analysis showed that the GATA domain positions correspond to motif 1 (**Figure 2C**).

The gene structures of *CmGATA* members are diverse, with exon numbers ranging from 2 to 11 and intron numbers ranging from 1 to 10 (**Figure 2D**). Members of the four groups exhibited conservation in the number of exons and introns. In groups I and II, except for *CmGATA5* and *CmGATA17*, which have 3 exons and 2 introns, the other 16 *CmGATA* genes each contained 2 exons and 1 intron. In group III, *CmGATA1*, *CmGATA3*, and *CmGATA7* each had 7 exons and 6 introns, while *CmGATA8* had 11 exons and 10 introns. The two CmGATA genes in group IV each had 8 exons and 7 introns. **Figure 2E** shows the correspondence between motifs, domains, and exon–intron structures with their respective colored rectangles. Overall, the diversity in protein motifs and gene structures suggests that *CmGATA* family members may have a wide range of functions in regulating the growth and development of melon.

### Localization, duplication, and Ka/Ks values of *CmGATA* members

**Figure 3:**
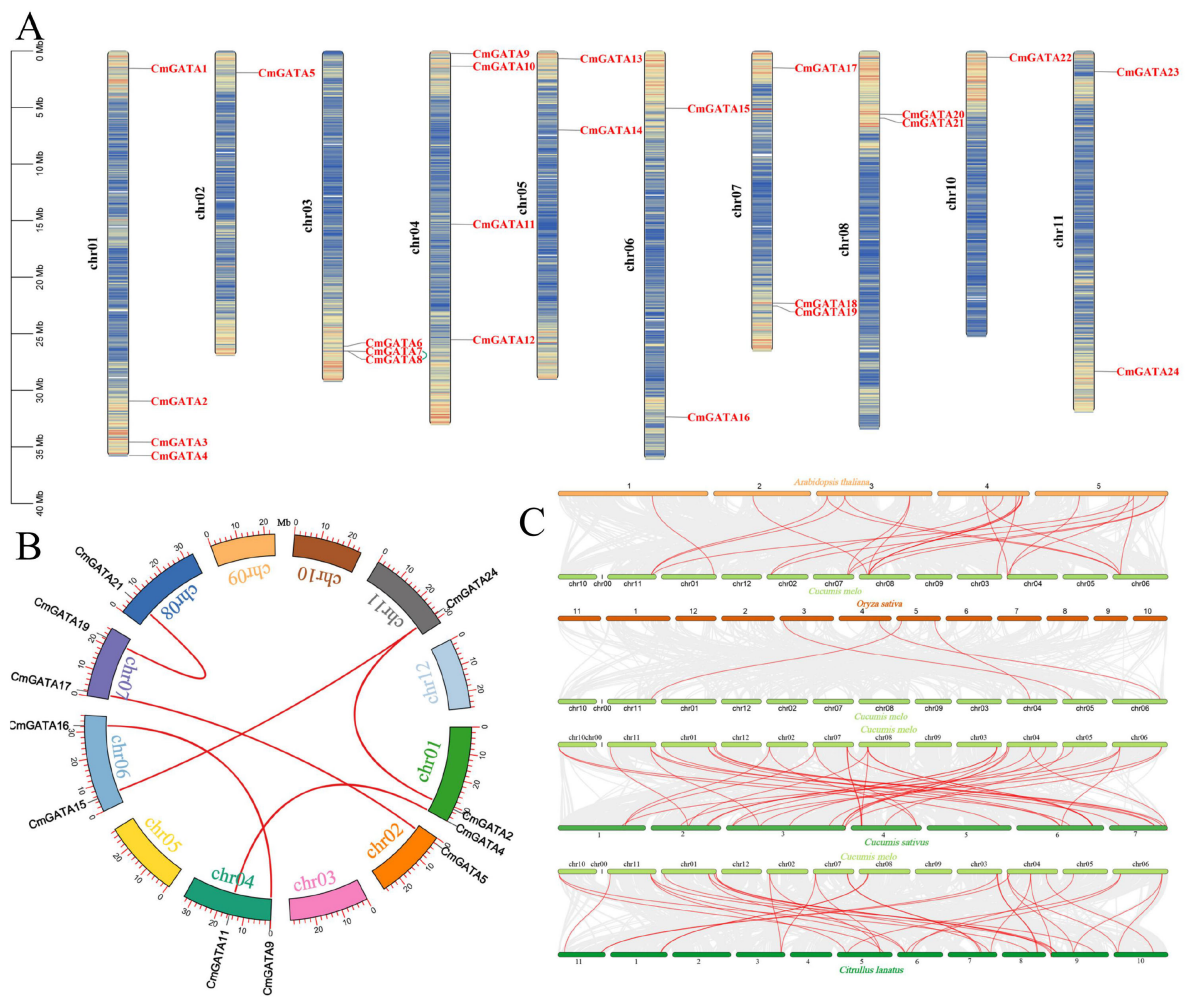
Analysis of *CmGATA* family members. **(A)** Gene chromosomal location analysis. The scale on the left represents the length of chromosomes, with ‘chr’ denoting the chromosome. Genetic intervals are set at 200 kb to calculate the gene density on each chromosome, with a gradient color from blue (low gene density) to red (high gene density) indicating this. Blank areas signify regions lacking gene distribution information. Green lines represent segmentally duplicated gene pairs. **(B)** Segmental duplication gene analysis. The arc shape represents chromosomes, with different colors corresponding to different chromosome numbers. The external red vertical scale indicates chromosome length. Red lines denote segmental gene pairs. **(C)** Analysis of *GATA* homologous gene pairs in melon, *A. thaliana*, *O. sativa*, *C. sativus*, and *C. lanatus*. Each horizontal line represents a chromosome, with numbers indicating the chromosome identifiers. Red lines denote the *GATA* homologous gene pairs.

CmGATA family members are distributed across ten chromosomes: chr01, chr02, chr03, chr04, chr05, chr06, chr07, chr08, chr10, and chr11, with respective member counts of 4, 1, 3, 4, 2, 2, 3, 2, 1, and 2 (**Figure 3A**). Additionally, *CmGATA* family members are predominantly located in regions of high gene density on the chromosomes. Six pairs of segmentally duplicated genes (*CmGATA2/CmGATA24*, *CmGATA4/CmGATA11*, *CmGATA5/CmGATA17*, *CmGATA9/CmGATA16*, *CmGATA15/CmGATA24*, and *CmGATA19/CmGATA21*) and one pair of tandemly duplicated genes (*CmGATA7/CmGATA8*) were identified, with segmental duplications distributed across chromosomes chr01, chr02, chr04, chr06, chr07, chr08, and chr11, and tandem duplications on chromosome chr03 (**Figures 3A, B**). Homologous gene pairs between melon and model dicot (*A. thaliana*) and monocot (*O. sativa*) species were identified. There were 21 homologous gene pairs identified between melon and *Arabidopsis*, and four between melon and rice. Furthermore, 38 and 34 pairs of homologous genes were identified between melon and cucumber, and melon and watermelon, respectively. Several genes, such as *CmGATA9*, *CmGATA15*, and *CmGATA11*, were found to have homologous relationships with multiple *GATA* genes in cucumber and watermelon, indicating a low degree of evolutionary divergence among these three closely related species (**Figure 3C**). Finally, Ka/Ks values were calculated for segmental, tandem, and homologous duplication gene pairs (**Supplementary Table 3**). The results indicated that these gene pairs all have Ka/Ks values less than 1, suggesting they are under strong purifying selection pressure. In summary, the analyses indicate that *CmGATA* family members are not distributed across all chromosomes, and gene duplication is a primary factor in the expansion of the family members, with a lower degree of divergence among dicot plants’ *GATA* genes during evolution.

### Promoter analysis of *CmGATA* family members

**Figure 4:**
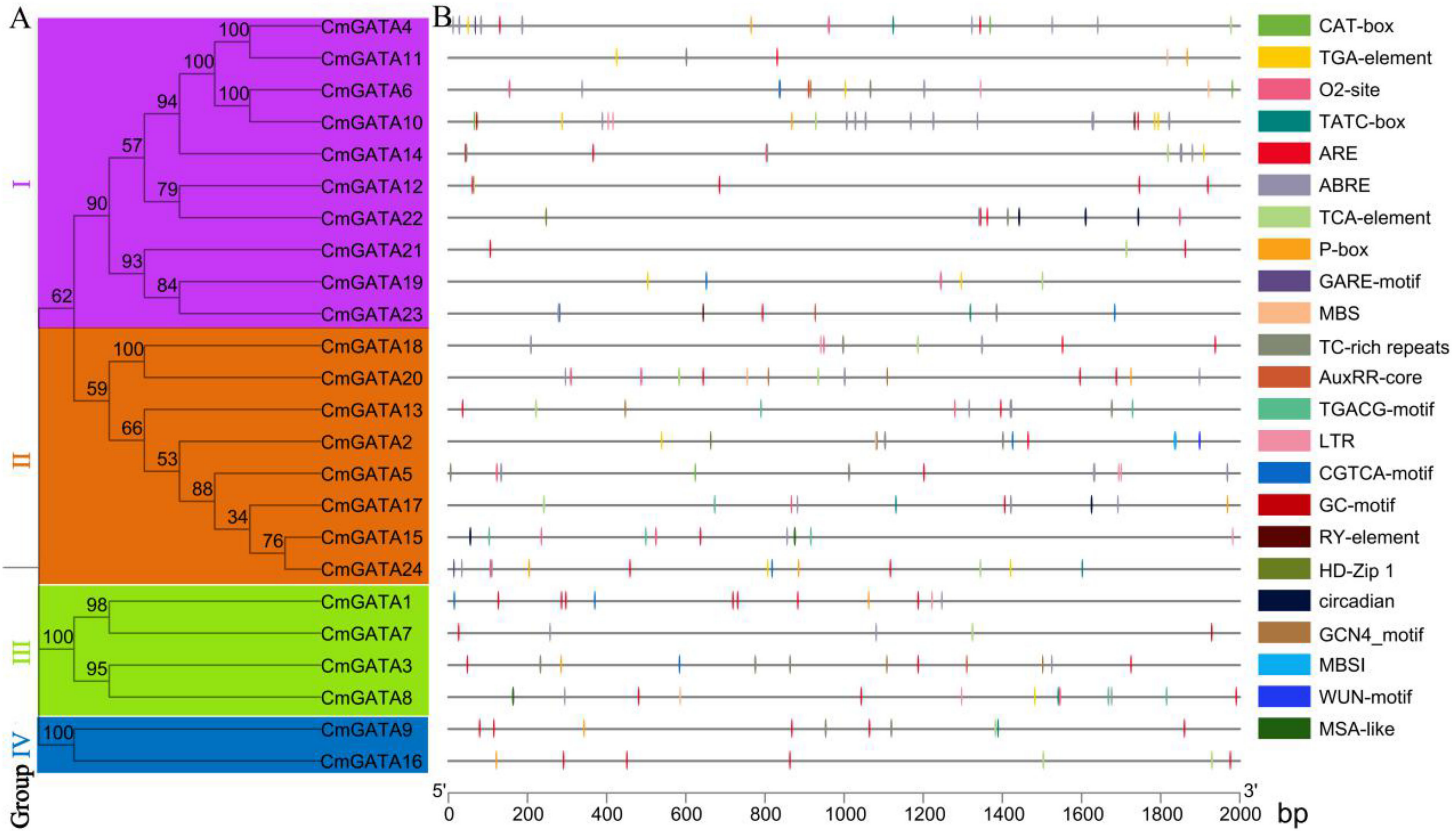
Analysis of *cis*-acting elements in *CmGATA* family members. **(A)** Neighbor-joining phylogenetic tree. **(B)** Distribution of *cis*-acting elements analysis.

There were 95 *cis*-acting elements identified in the *CmGATA* family members, 56 of which have specific functions. These elements include a large number of basic and light-responsive elements, as well as those related to abiotic and biotic stresses, phytohormone responsiveness, and plant growth and development (**Figure 4**; **Supplementary Table 4**). The elements associated with abiotic and biotic stresses include six types: TC-rich repeats, ARE, GC-motif, LTR, MBS, and WUN-motif. Phytohormone-responsive elements consist of nine types: P-box, TCA-element, TATC-box, TGA-element, ABRE, CGTCA-motif, TGACG-motif, AuxRR-core, and GARE-motif. Elements related to plant growth and development include eight types: CAT-box, RY-element, circadian, O2-site, MSA-like, GCN4_motif, HD-Zip 1, and MBSI. Overall, *CmGATA* family members likely play broad roles in the growth and development of melon, hormonal regulation, and stress response functions.

### Analysis of expression patterns in *CmGATA* family members

**Figure 5:**
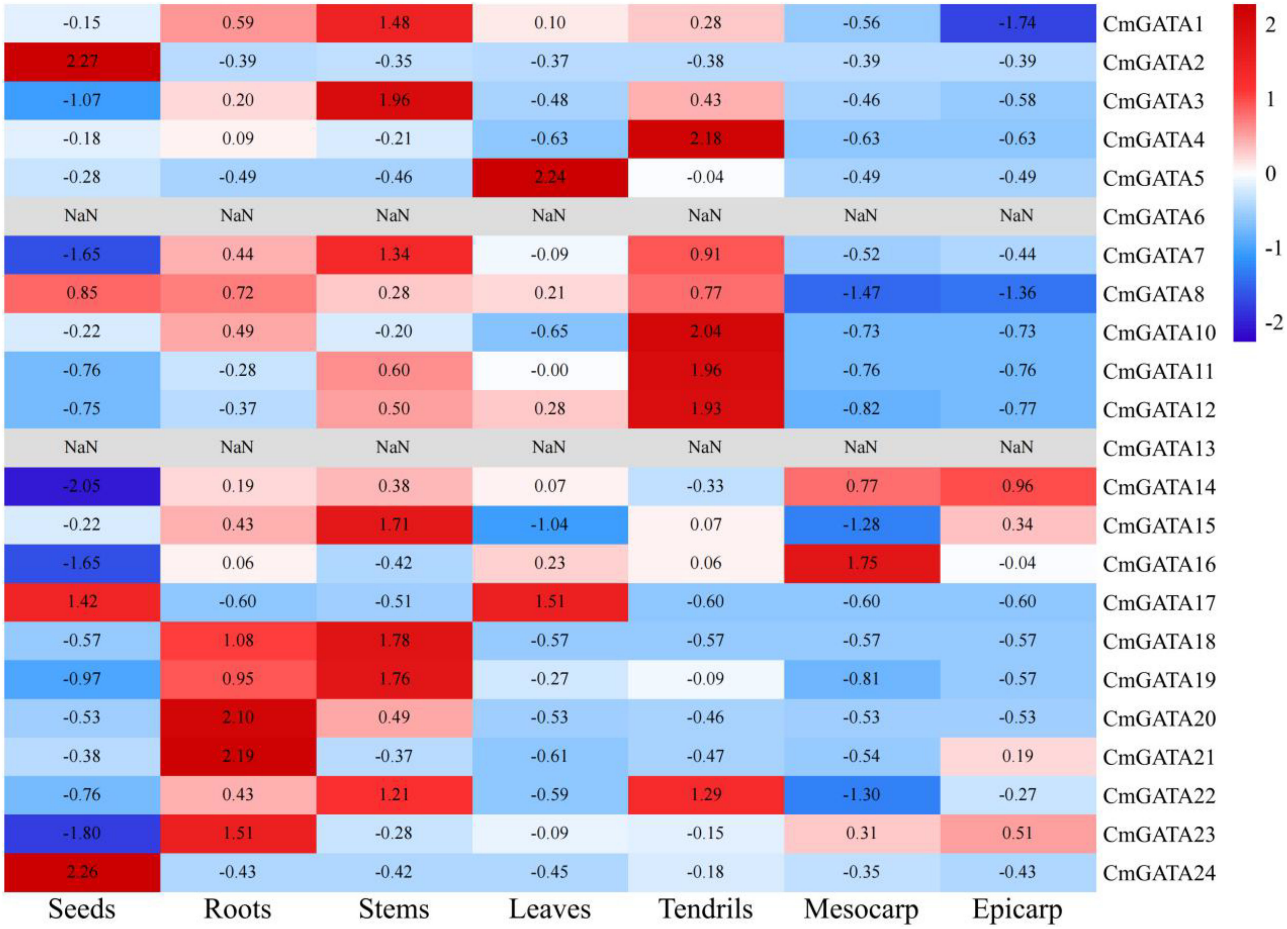
Analysis of expression patterns in *CmGATA* family members. Expression levels are normalized by row average, with a gradient from blue (low expression) to red (high expression).

FPKM values for 23 *CmGATA* family members were obtained. *CmGATA6* and CmGATA13 showed an FPKM of 0 across all tissues, and no FPKM value was obtained for *CmGATA9* (**Figure 5**; **Supplementary Table 5**). Four genes (*CmGATA2*, *CmGATA8*, *CmGATA17*, and *CmGATA24*) showed significant expression in seeds. Five genes (*CmGATA18*, *CmGATA19*, *CmGATA20*, *CmGATA21*, and *CmGATA23*) exhibited high expression levels in roots. Seven genes (*CmGATA1*, *CmGATA3*, *CmGATA7*, *CmGATA15*, *CmGATA18*, *CmGATA19*, and *CmGATA22*) displayed prominent expression in stems. Two genes, CmGATA5 and CmGATA17, had notable expression in leaves. Six genes (*CmGATA4*, *CmGATA7*, *CmGATA10*, *CmGATA11*, *CmGATA12*, and *CmGATA22*) exhibited significant expression in tendrils. *CmGATA14* and *CmGATA16* showed significant expression in the epicarp and mesocarp, respectively. Overall, the members of the *CmGATA* family exhibit significant tissue-specific expression patterns.

### Quantitative fluorescent expression analysis of *CmGATA* family members

**Figure 6:**
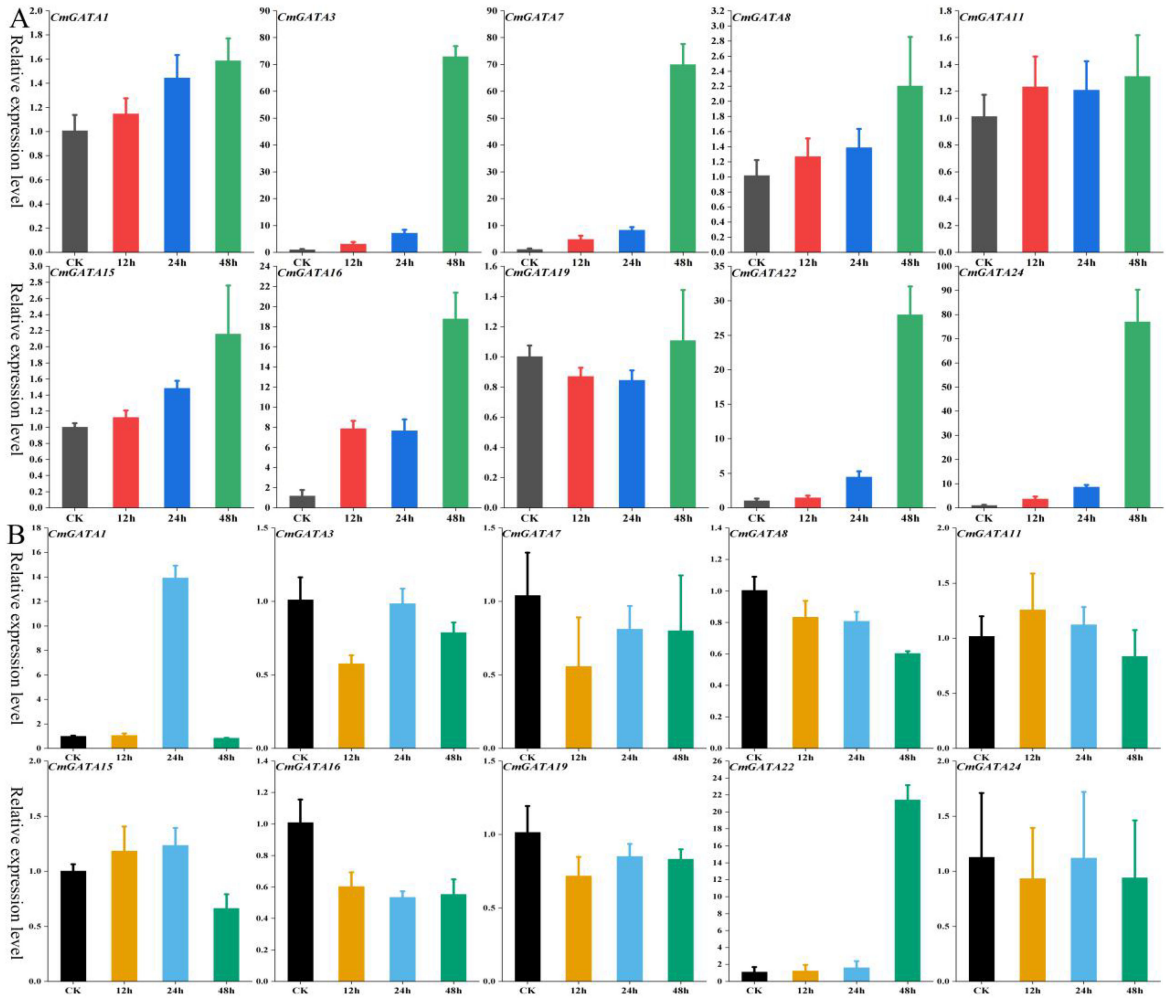
Fluorescent quantitative expression bar graph of *CmGATA* genes under drought and heavy metal lead stress. **(A)** Drought stress. **(B)** Heavy metal lead stress.

The expression level changes of 10 *CmGATA* genes in the ‘Super Sweet White Sugar Pot’ melon variety under drought stress and heavy metal lead stress were examined using qRT-PCR at four time points: 0 h, 12 h, 24 h, and 48 h. Under drought stress (**Figure 6A**), five genes (*CmGATA1*, *CmGATA8*, *CmGATA11*, *CmGATA15*, and *CmGATA19*) showed little change in expression levels over time. Conversely, *CmGATA3*, *CmGATA7*, *CmGATA16*, *CmGATA22*, and *CmGATA24* exhibited increasing expression levels over time, particularly at 48 h, where their expression levels sharply increased to 72.96, 69.99, 18.78, 27.99, and 77.09, respectively. Under heavy metal lead stress (**Figure 6B**), five genes (*CmGATA3*, *CmGATA7*, *CmGATA8*, *CmGATA16*, and *CmGATA19*) showed expression levels below the control (CK) at 12 h, 24 h, and 48 h. Three genes (*CmGATA11*, *CmGATA15*, and *CmGATA24*) exhibited expression levels slightly above the CK at some points. Notably, *CmGATA1* and *CmGATA22* showed significant increases in expression at 24 h and 48 h, reaching levels of 13.92 and 21.43, respectively. Overall, these findings indicate significant time-dependent changes in the expression levels of *CmGATA* genes in response to drought and heavy metal lead stress in melon.

**Figure 7:**
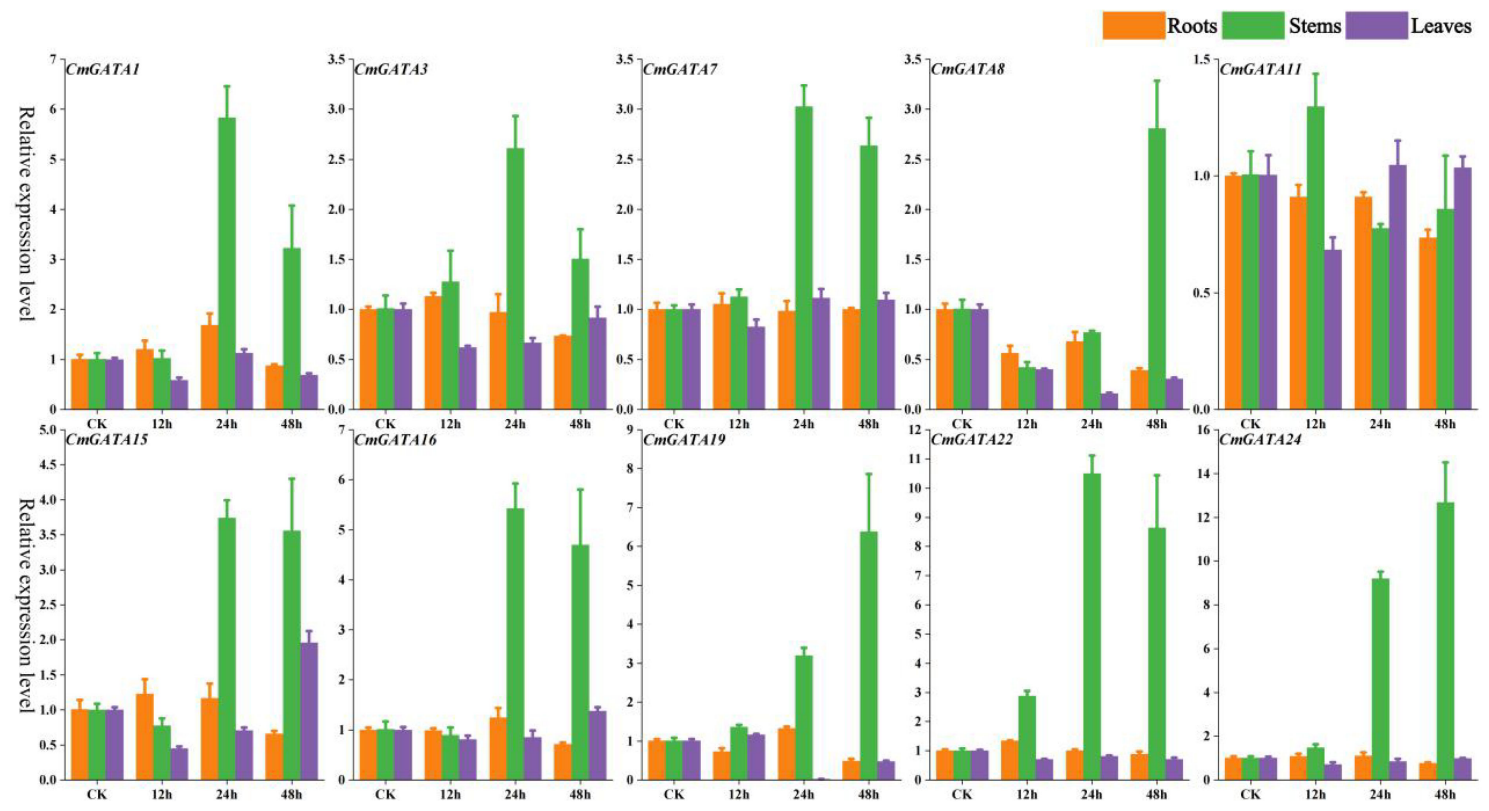
Fluorescent quantitative expression bar graph of *CmGATA* genes after infection by *Fusarium* wilt. Yellow bars represent roots, green bars represent stems, and purple bars represent leaves.

The expression changes of 10 *CmGATA* genes in roots, stems, and leaves of the ‘Super Sweet White Sugar Pot’ melon variety after *Fusarium* wilt infection at 0 h, 12 h, 24 h, and 48 h were also investigated using qRT-PCR (**Figure 7**). The expression levels of the 10 *CmGATA* genes changed little over the four time points in roots and leaves. Notably, except for *CmGATA11*, which showed no significant change in the stems, the other nine *CmGATA* genes displayed significantly increased expression levels at 24 h and 48 h, especially *CmGATA22* and *CmGATA24*, which reached expression levels of 10.49 and 9.21 at 24 h and 8.64 and 12.68 at 48 h, respectively. Thus, following *Fusarium* wilt infection, *CmGATA* genes may exhibit stronger resistance capabilities in the stems, while further investigation may be needed for roots and leaves.

### Protein interaction and target gene analysis

**Figure 8:**
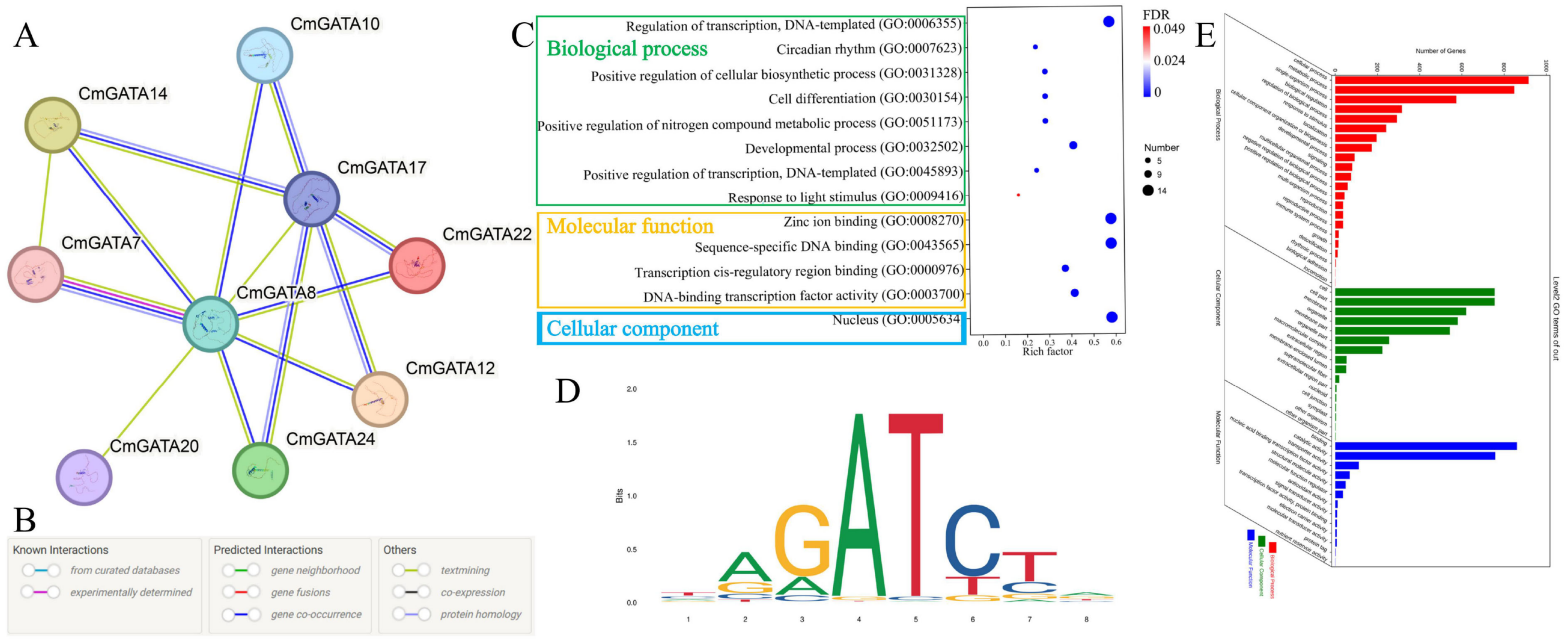
Protein interaction and target gene analysis. **(A)** Protein interaction network of CmGATA family members. **(B)** Meaning of different colored lines in the interaction network. **(C)** GO enrichment bubble chart of CmGATA family members. FDR stands for false discovery rate. **(D)** Binding site profile of the *Arabidopsis* GATA12 transcription factor. **(E)** Statistical results of GO enrichment for target genes.

Potential protein interactions among *CmGATA* members were predicted using the STRING tool based on the *A*. *thaliana* database (**Figures 8A, B**). Results indicated potential interactions among nine CmGATA members, with diverse interaction relationships, such as CmGATA20 only interacting with CmGATA8, while CmGATA14 potentially interacts with CmGATA7, CmGATA8, and CmGATA17 (**Supplementary Table 6**). *CmGATA8* was predicted to be a hub gene, interacting with eight other *CmGATA* members. Additionally, we performed GO enrichment analysis on the 24 CmGATA proteins (**Figure 8C**). The results showed enrichment in eight GO terms within the Biological Process category, including Regulation of transcription, DNA-templated (GO:0006355), Circadian rhythm (GO:0007623), and Positive regulation of cellular biosynthetic process (GO:0031328). In the Molecular Function category, four GO terms were enriched: Zinc ion binding (GO:0008270), Sequence-specific DNA binding (GO:0043565), Transcription *cis*-regulatory region binding (GO:0000976), and DNA-binding transcription factor activity (GO:0003700). Within the Cellular Component category, only one GO term, Nucleus (GO:0005634), was enriched.

Based on the binding motif map of *Arabidopsis* GATA12 transcription factor (MA1015.1) (**Figure 8D**), 2230 target genes were identified in the melon genome, including four types of matched sequences: TAGATCTA, TAGATCTG, CAGATCTA, and CAGATCTG (**Supplementary Table 7**). GO enrichment analysis was performed on these target genes (**Figure 8E**). Results showed that in Biological Processes, most target genes were enriched in cellular processes (GO:0009987), metabolic processes (GO:0008152), and single-organism processes (GO:0044699). In Cellular Components, most were enriched in cell (GO:0005623), organelle (GO:0043226), and membrane (GO:0016020). In Molecular Functions, they were predominantly involved in binding (GO:0005488), catalytic activity (GO:0003824), and transporter activity (GO:0005215). Overall, these results suggest potential regulatory interactions among *CmGATA* members that comprehensively modulate various responses in melon. The target gene results further indicate potential regulatory relationships with various gene types, thereby influencing corresponding growth and development processes.

### SSR loci and miRNA analysis of *CmGATA* family members

According to **Table 1**, *CmGATA2*, *CmGATA8*, and *CmGATA17* contain 9, 7, and 6 trinucleotide SSR loci, respectively, totaling 66 nucleotides. *CmGATA18* contains 4 complex nucleotide SSR loci, totaling 96 nucleotides. *CmGATA19* includes 26 mononucleotide SSR loci and 7 trinucleotide SSR loci, totaling 47 nucleotides. In total, 209 SSR loci were identified within the *CmGATA* family. Interactions between 120 miRNAs from the melon genome and all gene sequences were predicted. The results indicated that these 120 miRNAs formed 14,732 pairs of potential interactions with 5,194 genes. However, no potential interactions were found between these miRNAs and the 24 *CmGATA* genes (**Supplementary Table 8**).

**Table 1:** Prediction of SSR loci in *CmGATA* genes.

Gene name	SSR type	SSR type	SSR length	Start position	End position
CmGATA2	p3	(AAC)9	27	286	312
CmGATA8	p3	(GGC)7	21	139	159
CmGATA17	p3	(CTA)6	18	1127	1144
CmGATA18	p6	(CCTCTT)4	24	122	145
CmGATA19	p1	(T)26	26	1	26
CmGATA19	p3	(TGA)7	21	333	353

### *CmGATA* family member protein tertiary structure

**Figure 9:**
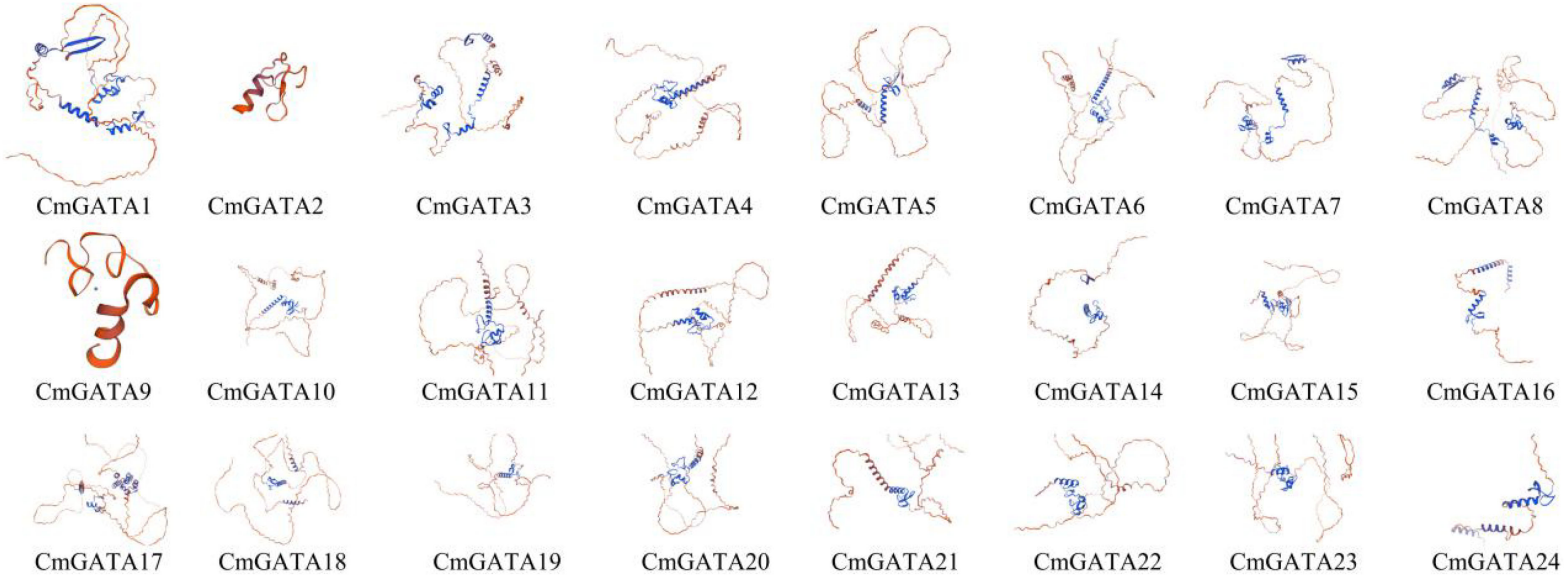
Predicted tertiary structure of CmGATA family member proteins.

The tertiary structures of the 24 CmGATA protein sequences were predicted using the SWISS-MODEL database and found to be highly similar to those of GATA transcription factors (**Figure 9**). Specifically, CmGATA2 and CmGATA9 proteins exhibited a high degree of structural similarity, while the remaining 22 CmGATA proteins demonstrated significant structural resemblance (**Figure 9**). In addition, the protein sequences of CmGATA2 and CmGATA9 are mainly composed of alpha-helix structures, while the secondary structures of the other 22 CmGATA proteins are primarily composed of alpha-helix and beta-sheet structures. The minimal variation in the tertiary structures among CmGATA proteins suggests that functional divergence within this family may have arisen from nucleotide changes during the course of evolution.

## Discussion

GATA transcription factors are evolutionarily conserved across animals, fungi, and plants (Block and Shapira, 2015; Schröder et al., 2023). They have been identified and studied in various plant species, such as *A*. *thaliana* (30) (Reyes et al., 2004), *O*. *sativa* (28) (Reyes et al., 2004), soybean (64) (Zhang et al., 2015), potato (49) (Yu et al., 2022), and wheat (79) (Feng et al., 2022), but there have been no reports on the *GATA* family in melon until now. In this study, 24 *CmGATA* family members were identified in the melon genome, a number close to that found in cucumber (26) (Zhang et al., 2021). The significant variation among *CmGATA* members in protein length, molecular weight, and theoretical isoelectric points indicates differentiation of *GATA* to adapt to environmental changes over the course of melon evolution. Subcellular localization predictions suggest that *CmGATA* members predominantly function in the nucleus, consistent with findings in soybean, cucumber, and potato.

Based on phylogenetic classification of *A*. *thaliana GATA* members, *CmGATA* family members were divided into four groups: group I, group II, group III, and group IV. This grouping is consistent with the phylogenetic classification results of most plant *GATA* family members, suggesting a relative stability of plant *GATA* members through evolutionary processes (Zhang et al., 2015; 2021). Multiple sequence alignment revealed that protein sequences in groups I, II, and III showed less conservation compared with group IV, which has high conservation, particularly at the LCNACG residues. Gene function is closely linked to the conserved motifs within the protein sequences. The 10 annotated motifs in *CmGATA* family members show that members within the same subfamily have more conserved motif types, indicating higher evolutionary conservation within these subfamilies. Notably, motif 1 corresponds to the GATA zinc finger, motif 3 to the CCT motif, and motif 7 to the tify domain. The CCT and TIFY domains play important roles in flowering and root development in *A. thaliana* (Liu et al., 2020; Zhang et al., 2023), suggesting that members of groups I and III may have similar regulatory functions in melon. Gene structure information provides clues to the evolution of gene family members. Consistent with most plant *GATA* family members (Zhang et al., 2015; Yu et al., 2022; Zhang et al., 2021), groups I and II have 2–3 exons, whereas groups III and IV have 7–11 exons. This further indicates the relative stability of *GATA* family members in plants.

*CmGATA* family members are unevenly distributed across 10 chromosomes (chr01 through chr11) and are predominantly located in regions of high gene density, likely resulting from various gene duplication events on the chromosomes. Gene duplication is a primary factor for gene family expansion and evolution, aiding species adaptation to environmental changes and maintaining normal life processes (Panchy et al., 2016). In *Sorghum bicolor* (Yao et al., 2023), *Fagopyrum tataricum* (Yao et al., 2022), *Brassica napus* (Zhu et al., 2020), and *C. sativus* (Zhang et al., 2021), *GATA* family members were found to have 16, 8, 82, and 6 pairs of segmentally duplicated genes, respectively. In the *CmGATA* family, six pairs of segmentally duplicated genes and one pair of tandemly duplicated genes were identified, highlighting gene duplication as the main factor for the expansion of the melon *GATA* family. The Ka/Ks values, which are effective for studying the evolutionary selection of duplicated genes, were found to be less than 1 for segmental, tandem, and homologous duplications, indicating these duplicated genes predominantly undergo purifying selection and have highly conserved functions.

*Cis*-acting elements within the promoter regions can regulate gene expression. The presence of these elements indicates that *CmGATA* family members are involved in various functions such as melon growth and development, stress response, and hormone regulation. Investigating gene expression during tissue development and under adverse environmental conditions is crucial for understanding the molecular mechanisms of biological development (Page and Minocha, 2005). *GATA* members in species like wheat, sorghum, and canola exhibit significant expression characteristics in roots, leaves, stems, and various floral organs. Based on transcriptomic data, FPKM values for *CmGATA* family members in seven different tissues: seeds, roots, stems, leaves, tendrils, mesocarp, and epicarp were obtained. The expression patterns show that *CmGATA* members have widespread significance across different melon tissues, with notable tissue-specific expression, suggesting that *CmGATA* members are broadly involved in the growth and development processes of different melon tissues.

To date, the *GATA* family has been studied in dozens of species, and their potential functions have been explored. The overexpression of the *OsGATA8* gene in rice significantly bolsters its drought tolerance (Gupta et al., 2017), while the enhancement of drought resistance in tomatoes is achieved through the overexpression of the *SlGATA17* gene (Zhao et al., 2021). Similarly, the transgenic *Arabidopsis*, with the overexpression of the *BdGATA13* gene exhibits improved drought tolerance (Guo et al., 2021b). In *Ophiorrhiza pumila*, the *OpGATA7* gene has been implicated in the biosynthesis of camptothecin in roots (Shi et al., 2022). In *Setaria italica*, multiple *SiGATA* genes were found to be induced under eight different abiotic stresses (acid, alkali, NaCl, PEG, dark, flooding, heat, and cold), suggesting a potential role in enhancing resistance to abiotic stresses (Lai et al., 2022). In *P. bretschneideri*, *PbGATA* genes were induced following exogenous hormone treatments with salicylic acid (SA), methyl jasmonate (MeJA), and abscisic acid (ABA), indicating these genes may play significant roles in hormone signaling pathways (Manzoor et al., 2021). In *T. aestivum*, *TaGATA* genes were found to enhance drought and salt tolerance through interactions between the DNA-binding motif of GATA transcription factors and the light morphogenesis-related protein TaCOP9-5A (Du et al., 2023). In *Alternaria alternata*, *GATA* genes are important regulatory factors involved in fungal development, conidiation, detoxification of reactive oxygen species, and pathogenicity (Chen et al., 2021).

Quantitative fluorescence results show significant temporal expression gradients for 10 *CmGATA* genes under melon drought stress, particularly at the 48-h mark, with significant increases in the expression levels for *CmGATA3*, *CmGATA7*, *CmGATA16*, *CmGATA22*, and *CmGATA24*. *CmGATA* genes may regulate melon’s resistance to drought stress, with the peak activity likely occurring around 48 hours after stress onset.

Studies on the regulatory relationship between *GATA* and heavy metals have not yet been reported. Quantitative fluorescence shows that the expression levels of eight *CmGATA* genes under heavy metal lead stress in melon change minimally at different time points, with only *CmGATA1* and *CmGATA22* showing significant increases at specific times. *CmGATA* genes may have the potential to regulate melon’s response to heavy metal stress. However, further in-depth studies are needed to confirm this. Research on *GATA* regulation of species resistance to abiotic stress has achieved substantial results, such as *ZmGATA37* showing significantly enhanced expression in maize under attack by smut disease and aphids (Hu et al., 2023), and the cucumber *Cs5G622830* (GATA) gene showing significantly increased expression after treatment with downy mildew, powdery mildew, and root-knot nematode infections (Zhang et al., 2021). In grapes, most *VvGATA* genes were found to potentially play roles in resistance to downy mildew (Chen et al., 2022).

Quantitative fluorescence shows that the expression levels of 10 *CmGATA* genes in melon under *Fusarium* wilt infection are low at four time points in roots and leaves, while the stems exhibit significant temporal gradient expression changes. Nine *CmGATA* genes showed a significant increase in expression levels in melon stems at 24 h and 48 h postinfection, especially *CmGATA22* and *CmGATA24*. *CmGATA* genes may play a potential role in regulating melon’s defense against pathogen infection, exhibiting different response characteristics in various tissues after infection. Notably, *CmGATA22* may have the potential to regulate melon’s resistance to both biotic and abiotic stresses, meriting further in-depth investigation. In summary, the expression characteristics of *CmGATA* in various tissues were explored and quantitative fluorescence techniques were used to study the expression level changes of 10 *CmGATA* genes in melon under drought stress, heavy metal lead stress, and *Fusarium* wilt infection at four time points. These results can only serve as references, and further research is needed to understand the specific functions of these genes.

Transcription factors often regulate various response processes through protein–protein interactions. Potential interactions among nine *CmGATA* members were predicted, providing a basis for further exploration of CmGATA interactions. Transcription factors regulate gene expression by binding to specific *cis*-regulatory sequences within the promoters of target genes. However, studies on GATA transcription factor target genes in plants are relatively limited. This study utilized the binding motif map of the *A*. *thaliana* GATA12 transcription factor (MA1015.1) to identify 2,230 target genes in the melon genome. The predicted GO functions and structural domains of these target genes provide a foundation for future research into the interactions between melon GATA transcription factors and their target genes.

## Conclusion

In this study, 24 *CmGATA* genes were identified in the melon DHL92 v4.0 genome, and their physicochemical properties, phylogenetics, gene structure, protein motifs, gene duplication, and expression patterns were analyzed. The phylogenetic analysis divided the *CmGATA* family into four groups, with members within the same group showing high conservation in gene structure and protein motifs. Gene duplication was identified as one of the main factors contributing to the expansion of the *CmGATA* family. Finally, using quantitative fluorescence techniques, the expression patterns of ten *CmGATA* genes in melon under conditions of drought stress, heavy metal lead stress, and *Fusarium* wilt infection at four time points were examined. The results of this study provide significant insights into the characteristics and functions of the *CmGATA* family members.

## Data Availability

The original contributions presented in the study are included in the article/**Supplementary Material**. Further inquiries can be directed to the corresponding author.
