# Supplementary figures and images for: Genome-wide identification of the GATA gene family in melon (Cucumis melo) and analysis of their expression characteristics under biotic and abiotic stresses
Source: Front Plant Sci. 2024 Sep 13;15:1462924. doi: 10.3389/fpls.2024.1462924 (PMC11427367; doi:10.3389/fpls.2024.1462924)

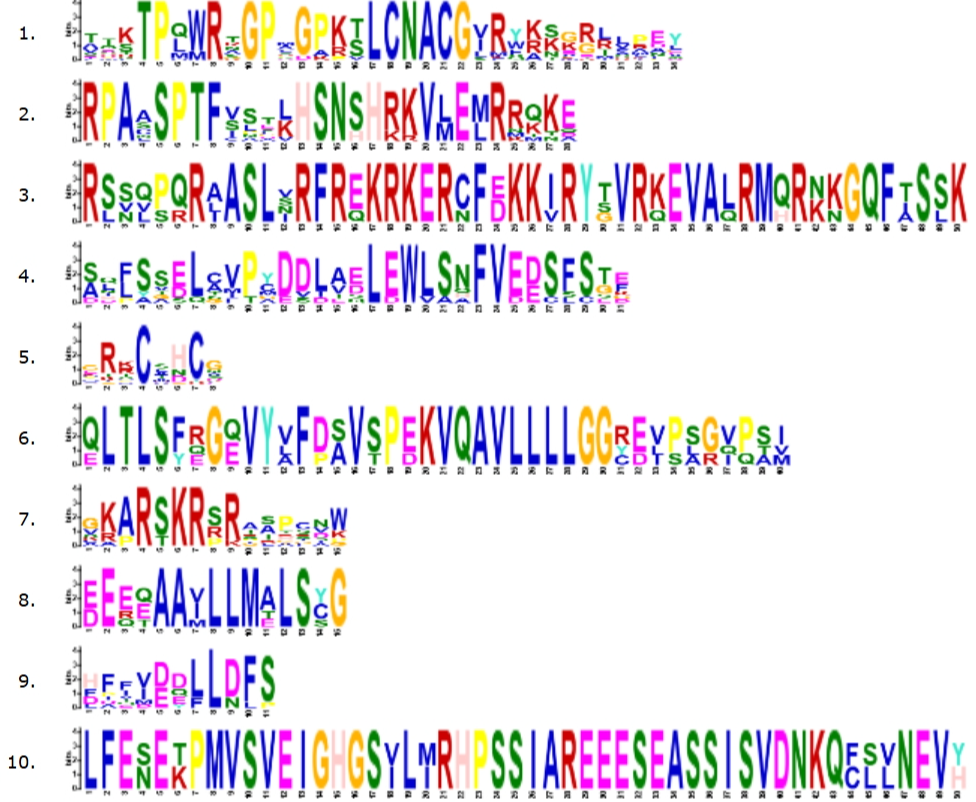

Supplement: Supplementary file 1 [file DataSheet1.zip › Supplementary files/Figures/Supplementary figure2.png]
