# Supplementary material for: Genome-wide identification of the GATA gene family in melon (Cucumis melo) and analysis of their expression characteristics under biotic and abiotic stresses
Source: Front Plant Sci. 2024 Sep 13;15:1462924. doi: 10.3389/fpls.2024.1462924 (PMC11427367; doi:10.3389/fpls.2024.1462924)
Supplement: Supplementary file 2 [file DataSheet2.zip › Supplementary/Supplementary Figures.docx]

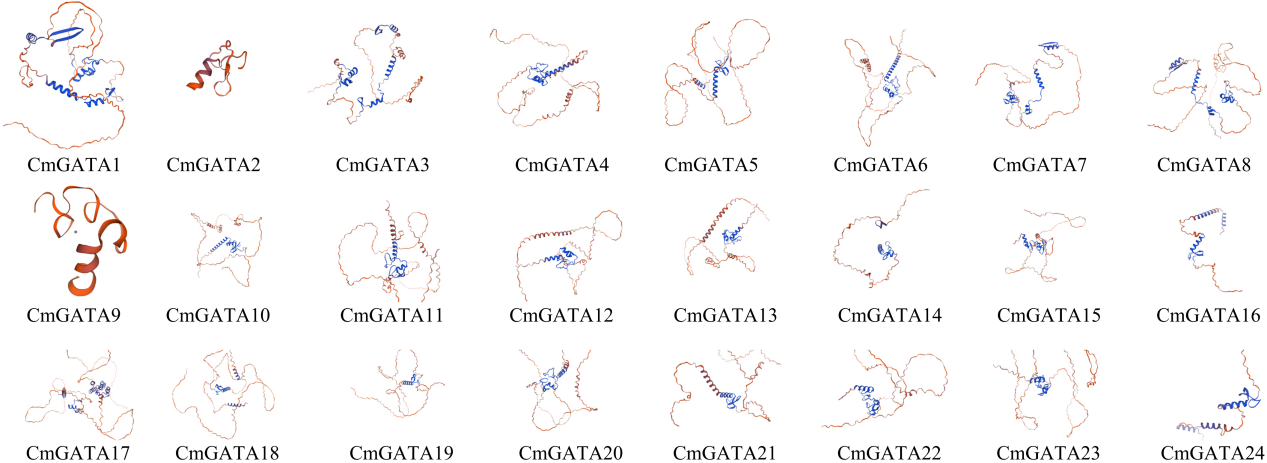


Supplementary Figure 1: Tertiary structures of CmGATA family member proteins.


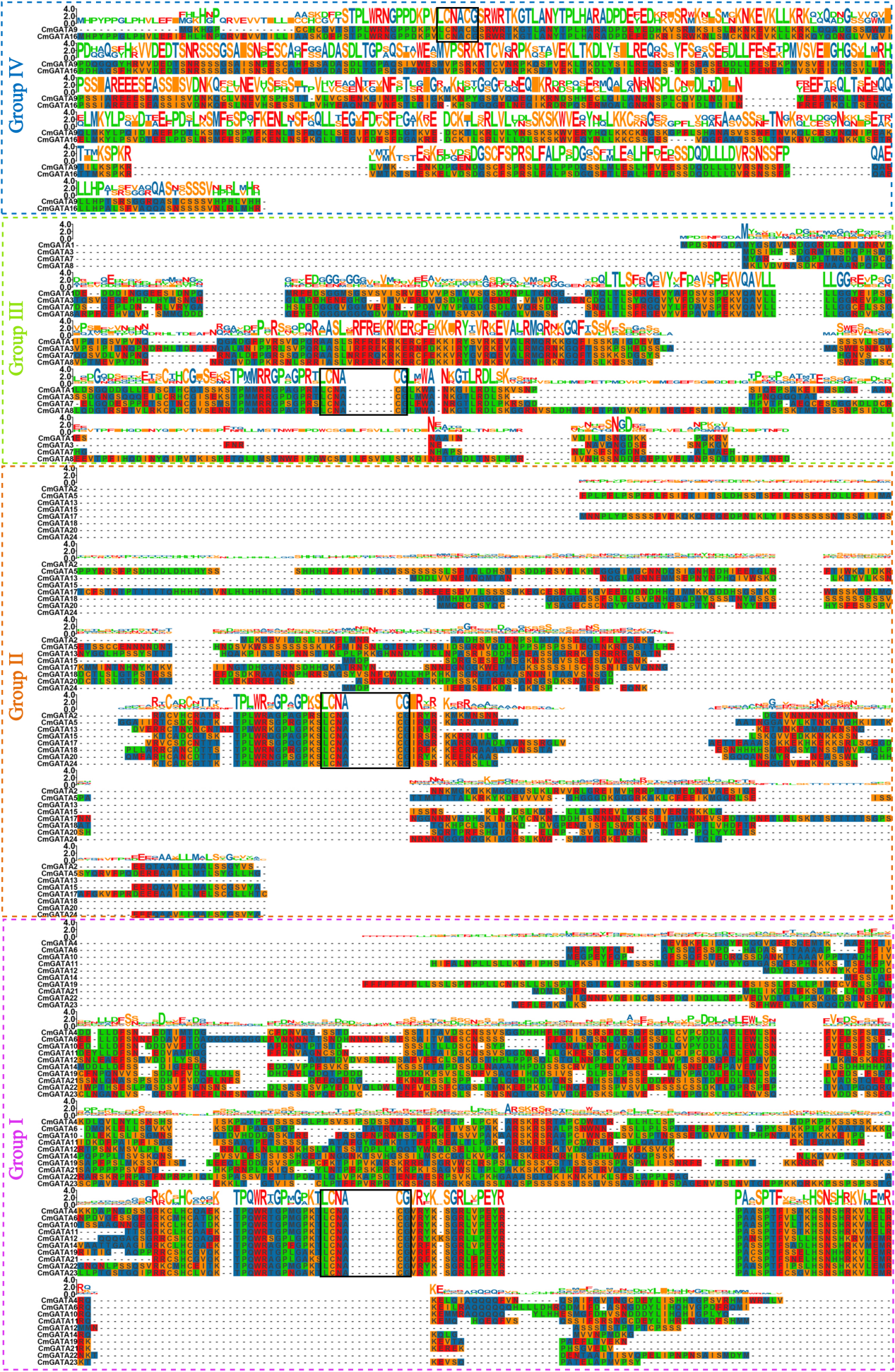


Supplementary Figure 2: Multiple sequence alignment of CmGATA family member proteins.


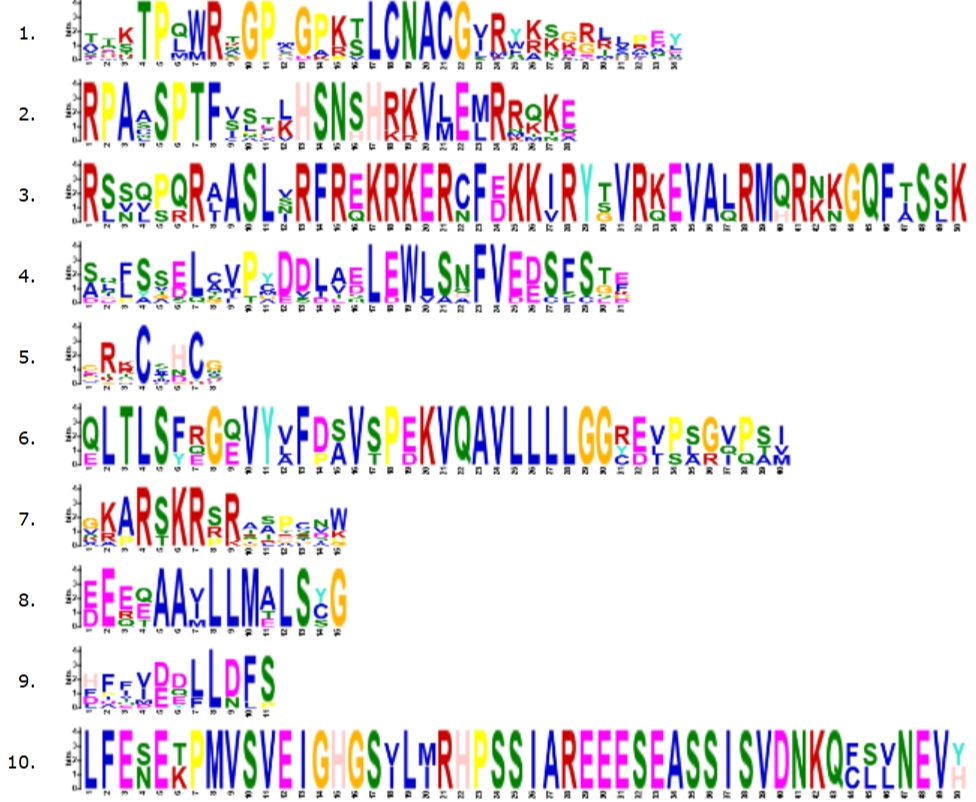


Supplementary Figure 3: Conserved amino acid regions and types corresponding to the 10 motifs.
