# Supplementary material for: Genome-wide identification of the GATA gene family in melon (Cucumis melo) and analysis of their expression characteristics under biotic and abiotic stresses
Source: Front Plant Sci. 2024 Sep 13;15:1462924. doi: 10.3389/fpls.2024.1462924 (PMC11427367; doi:10.3389/fpls.2024.1462924)
Supplement: Supplementary file 3 [file DataSheet3.zip › Supplementary Figures8.5.docx]

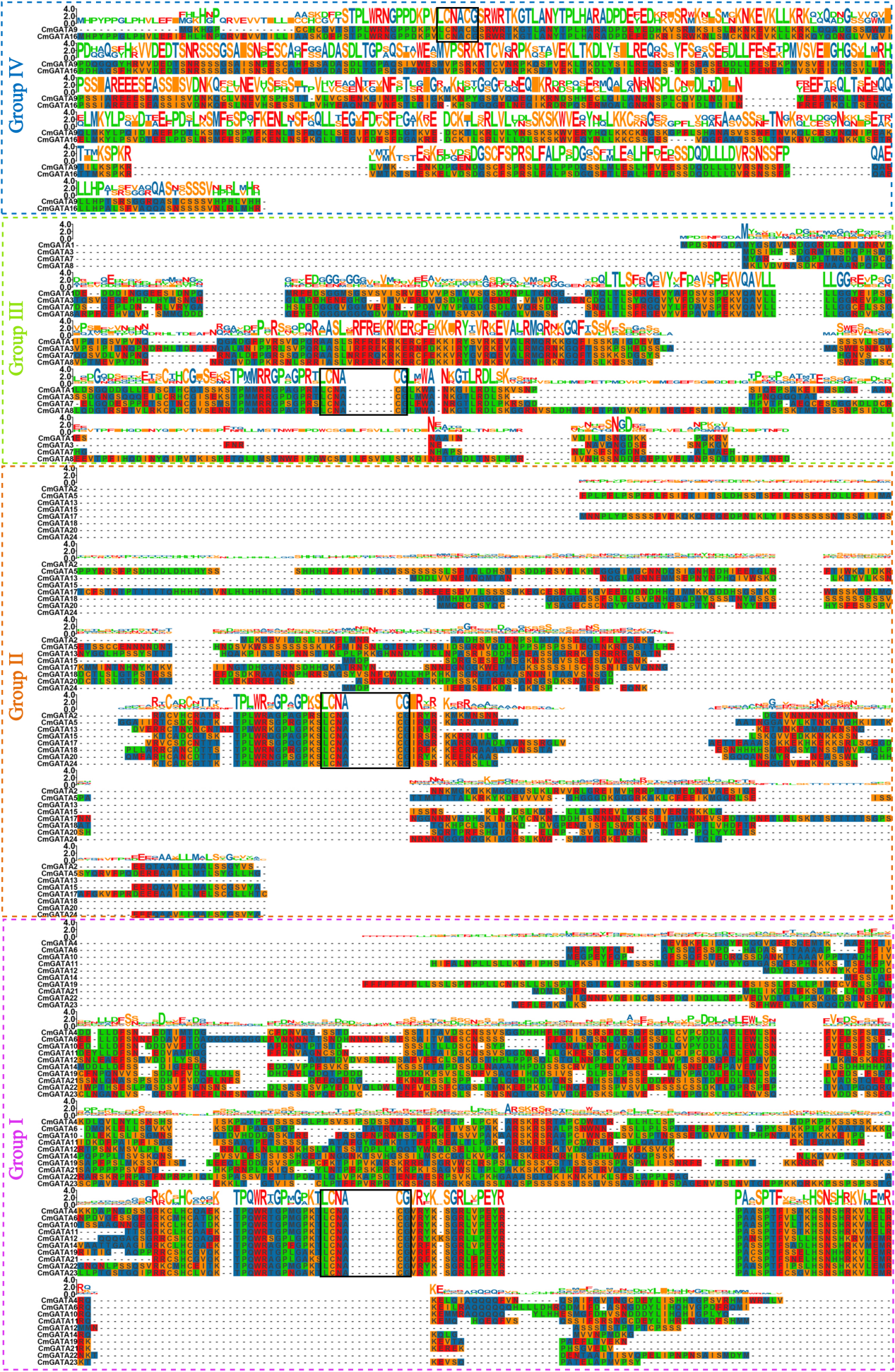


Supplementary Figure 1: Multiple alignment of protein sequences of CmGATA family members.


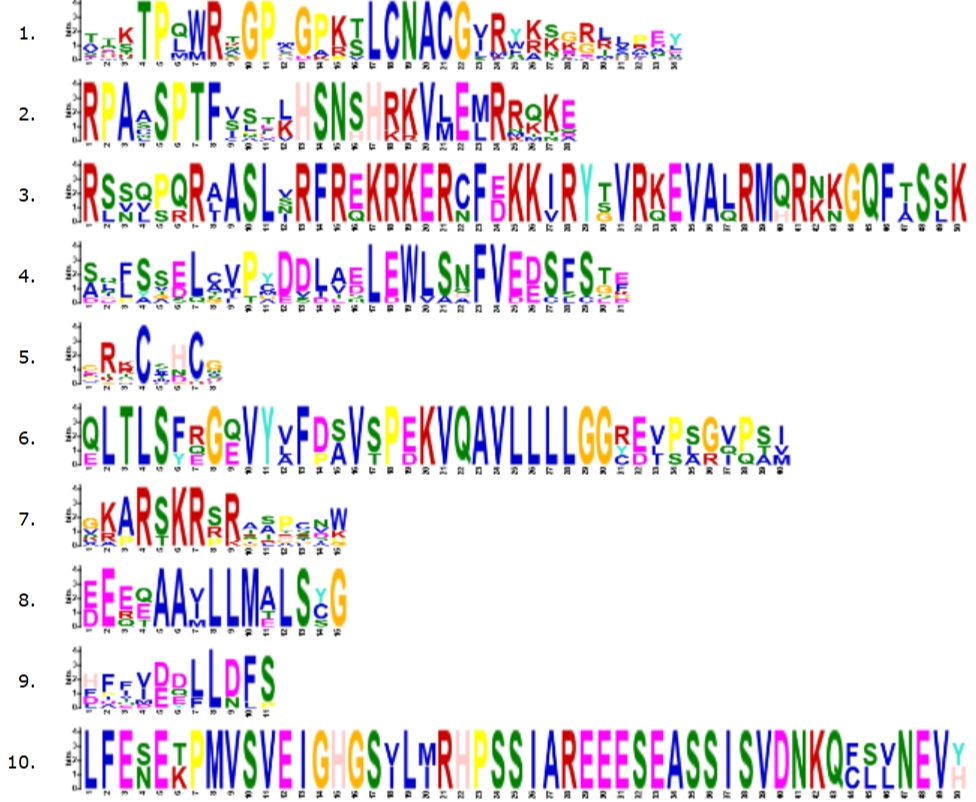


Supplementary Figure 2: 10 conserved amino acid regions and types corresponding to Motifs.
