# Supplementary material for: Genome-wide identification of the GATA gene family in melon (Cucumis melo) and analysis of their expression characteristics under biotic and abiotic stresses
Source: Front Plant Sci. 2024 Sep 13;15:1462924. doi: 10.3389/fpls.2024.1462924 (PMC11427367; doi:10.3389/fpls.2024.1462924)
Supplement: Supplementary file 1 [file Supplementary figure 1.pdf › Supplementary files/Figures/Supplementary figure 1.pdf]

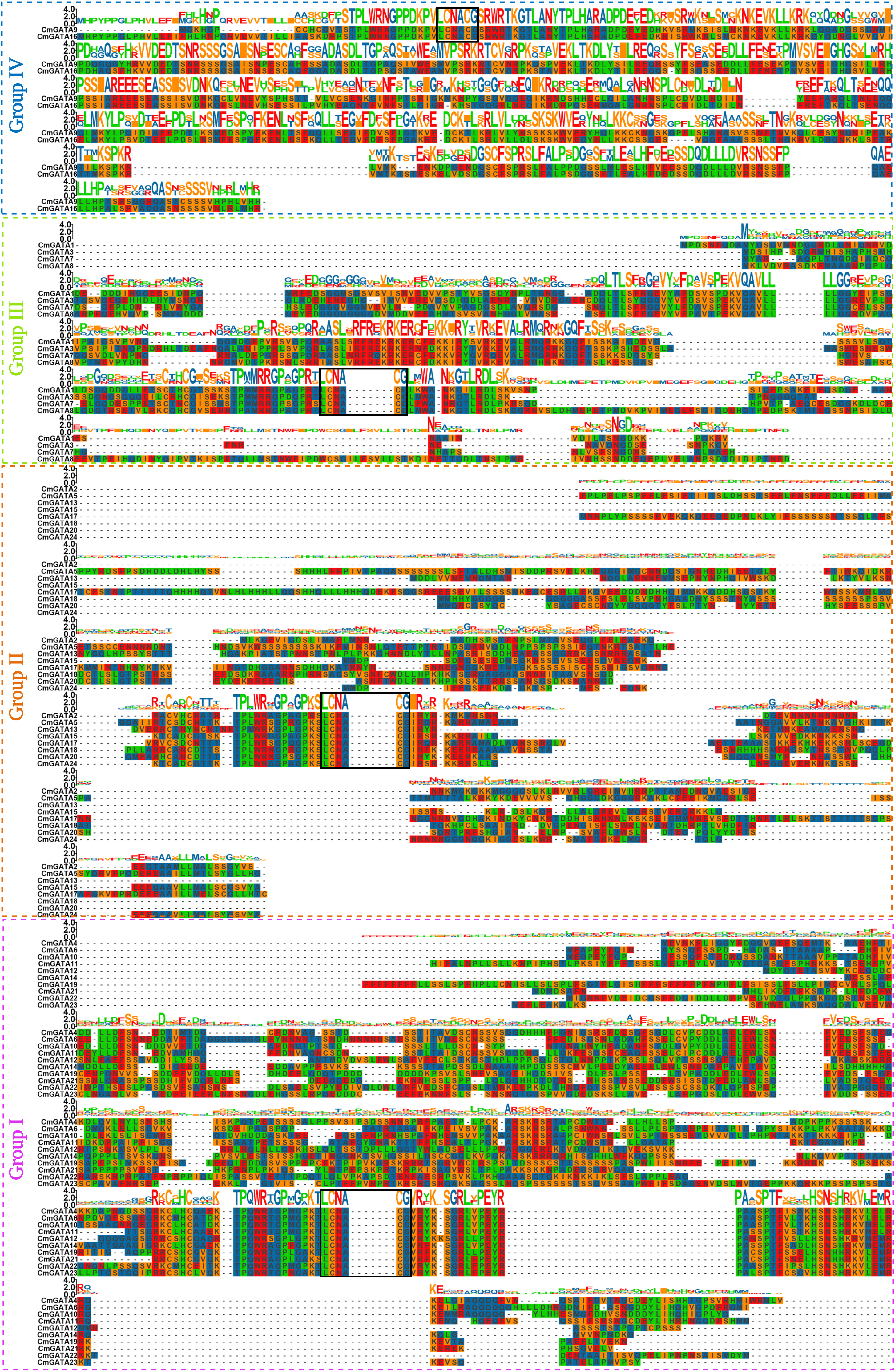
Group IV
CmGATA9
CmGATA16
Group III
CmGATA1
CmGATA3
CmGATA7
CmGATA8
Group II
CmGATA2
CmGATA5
CmGATA13
CmGATA15
CmGATA17
CmGATA18
CmGATA20
CmGATA24
Group I
CmGATA4
CmGATA6
CmGATA10
CmGATA11
CmGATA12
CmGATA14
CmGATA19
CmGATA21
CmGATA22
CmGATA23
